# Verapamil chronicles: advances from cardiovascular to pancreatic β-cell protection

**DOI:** 10.3389/fphar.2023.1322148

**Published:** 2023-11-27

**Authors:** Hossein Arefanian, Lubaina Koti, Sardar Sindhu, Rasheed Ahmad, Ashraf Al Madhoun, Fahd Al-Mulla

**Affiliations:** ^1^ Immunology and Microbiology Department, Dasman Diabetes Institute, Kuwait City, Kuwait; ^2^ Department of Genetics and Bioinformatics, Dasman Diabetes Institute, Kuwait City, Kuwait; ^3^ Animal and Imaging Core Facility, Dasman Diabetes Institute, Kuwait City, Kuwait

**Keywords:** Verapamil, clinical trial, diabetes, T1D, T2D, DM, β-cell

## Abstract

Verapamil is a well-known drug used for treating angina and hypertension. Emerging data from current clinical trials suggest that this calcium channel blocker has a potential benefit for pancreatic β-cells through the elevation and sustenance of C-peptide levels in patients with diabetes mellitus (DM). This is intriguing, given the fact that the current therapeutic options for DM are still limited to using insulin and incretins which, in fact, fail to address the underlying pathology of β-cell destruction and loss. Moreover, verapamil is widely available as an FDA-approved, cost-effective drug, supported also by its substantial efficacy and safety. However, the molecular mechanisms underlying the β-cell protective potentials of verapamil are yet to be fully elucidated. Although, verapamil reduces the expression of thioredoxin-interacting protein (TXNIP), a molecule which is involved in β-cell apoptosis and glucotoxicity-induced β-cell death, other signaling pathways are also modulated by verapamil. In this review, we revisit the historical avenues that lead to verapamil as a potential therapeutic agent for DM. Importantly, this review provides an update on the current known mechanisms of action of verapamil and also allude to the plausible mechanisms that could be implicated in its β-cell protective effects, based on our own research findings.

## 1 Introduction

Diabetes mellitus (DM), a serious chronic metabolic disorder, is emerging as a global threat, with devastating human health, economic and social implications, and a negative impact on patients’ life expectancy and quality. According to the American Diabetes Association (ADA), diabetes is categorized into four types: type 1 diabetes (T1D); type 2 diabetes (T2D); gestational diabetes mellites (GDM); and specific sub-types of diabetes caused by monogenic syndromes, pancreatitis, or drug-induced diabetes (American Diabetes Association Professional Practice Committee, 2022).

This review focuses on T1D and T2D, which are heterogenous diseases that differ in etiology, clinical presentation, and progression. Both T1D and T2D are multifactorial polygenic metabolic disorders influenced by environmental, genetic, and/or pathophysiological factors (Tuomi, 2005; Ndisang et al., 2017).

T1D is a chronic disease caused by the autoimmune damage to pancreatic β-cells, resulting in insulin deficiency, hyperglycemia and various health complications (Kahanovitz et al., 2017). T1D is typically manifested during childhood and adolescence, but it can also develop in adults (DiMeglio et al., 2018). Islet autoimmunity is the main cause of T1D which is developed by a mechanism that remains to be clearly elucidated. Recent evidence shows that a combination of the genetic predisposition and environmental factors causes T1D, with notable increases in disease incidences over the past few years (Rewers and Ludvigsson, 2016). Management of T1D requires the lifelong insulin therapy to regulate blood sugar levels. Along with insulin, a healthy diet, regular physical activity, and regular blood glucose monitoring are crucial for effective diabetes management. In addition, pancreas and/or islet transplantations are the other therapeutic strategies to consider (Walker et al., 2022).

The incidence of T2D is increasing globally, driven by a multitude of factors such as aging, urbanization, and unhealthy lifestyles. Although T2D is the most common form of diabetes that primarily affects adults, concerningly, the disease prevalence among the children and adolescents has also been notably escalating over the past two decades (Chen et al., 2011). T2D is largely caused by development of peripheral tissues insulin resistance, as well as by lack of insulin production and secretion due to the impairment and a massive loss of functional pancreatic β-cells, especially in advanced stages of the disease. T2D is a complex multifactorial disease which sets stage for the development of several metabolic alterations including hyperglycemia, dyslipidemia, and a distinct bioenergetic impairment (Okon et al., 2007; D'Alessio, 2011; Lin and Sun, 2010). The exact cause of T2D is complex and involves a combination of genetic and epigenetic factors (Galicia-Garcia et al., 2020). Genetics plays a significant role as the individuals with a family history of T2D are at an increased risk of developing the condition (Fuchsberger et al., 2016). Epigenetic or the environmental factors, such as obesity, a sedentary lifestyle or physical inactivity coupled with overnutrition, especially intake of diets rich in unhealthy saturated fats and/or over-loaded with sugars, also contribute to the development of T2D (Ferrannini et al., 1997; Hu et al., 2001). Given this complex etiology, management of T2D typically involves several lifestyle modifications, such as healthy eating, regular physical activity, and a number of medications including metformin, sulfonylureas, and/or incretins as well as insulin injections.

Despite these differences, T1D and T2D may also share some common features, such as hyperglycemia and impaired pancreatic β-cell function (Vladu et al., 2017), and their association with life-threating complications, such as atherosclerosis, neuropathy, retinopathy, and nephropathy (Cade, 2008). The current therapeutic approach for the treatment of T1D may increase the risk of complications including hypoglycemia (in case of insulin injections) and immunosuppression (in case of the chronic use of immunosuppressive drugs to maintain transplanted pancreas and/or islets) (Hirshberg et al., 2003; Kolb et al., 2020). While for T2D management, drugs that mimic the dual action of two main incretin hormones, glucagon-like peptide-1 (GLP-1) and glucose-dependent insulinotropic polypeptides (GIP) receptor agonists (RAs) have shown significant benefits for glucose control, weight reduction, and positive effects on β-cell function and possible β-cell preservation (Kutoh, 2011; Min and Bain, 2021). Nonetheless, these therapies also have some underlying disadvantages, as, for example, the GLP-1/GIP RAs and insulin injections which are administered subcutaneously may face aversion from patients due to concerns of invasiveness, pain, and discomfort (Kruger et al., 2015). GLP-1/GIP RAs, owing to their type and demand, are currently expensive, inaccessible, and unfeasible for patients who are looking to initiate or continue treatment, adding to the already overwhelming burden of the disease (Whitley et al., 2023). To overcome these limitations, it is imperative to develop novel therapeutic molecules or repurpose the existing drugs that prevent the progression of T1D and T2D by preserving pancreatic β-cell function, whilst also catering to the concerns of cost-effectiveness, clinical complications, and availability.

One such repurposed drug is verapamil, a calcium channel blocker (CCB) approved by the Food and Drug Administration (FDA) in the late 1990s for the treatment of hypertension, angina, and arrythmia (Verapamil Infarction Trial II--DAVIT II, 1990). It was found that oral verapamil administration lowered the risk of developing new-onset diabetes in various studies and lowered fasting glucose levels in patients with diabetes (Cooper-DeHoff et al., 2006a). This narrative review chronicles the journey of verapamil, originally approved for hypertension management, as it ventures into the realm of treating DM. By delving into the intricate mechanisms through which verapamil safeguards pancreatic β-cells and enhances glucose homeostasis, this review sheds light on its potential as a therapeutic option for diabetes treatment. This review also discusses *in vitro* and *in vivo* studies and clinical trials, aiming at exploring verapamil therapy, along with future, unexplored avenues in the context of precision medicine options pertaining to verapamil treatment. Through this narrative review of the literature, the aim is to shed light on the fact that the initial clinical trials highlighted in this review primarily aimed to study the cardioprotective effects of verapamil and the secondary endpoints of these trials included its glucometabolic benefits. By highlighting these secondary end point benefits, the scientists involved in future clinical trials regarding various diseases are suggested to pay attention to these critical nuances in trial outcomes.

## 2 Structural and functional attributes of Verapamil

**FIGURE 1:**
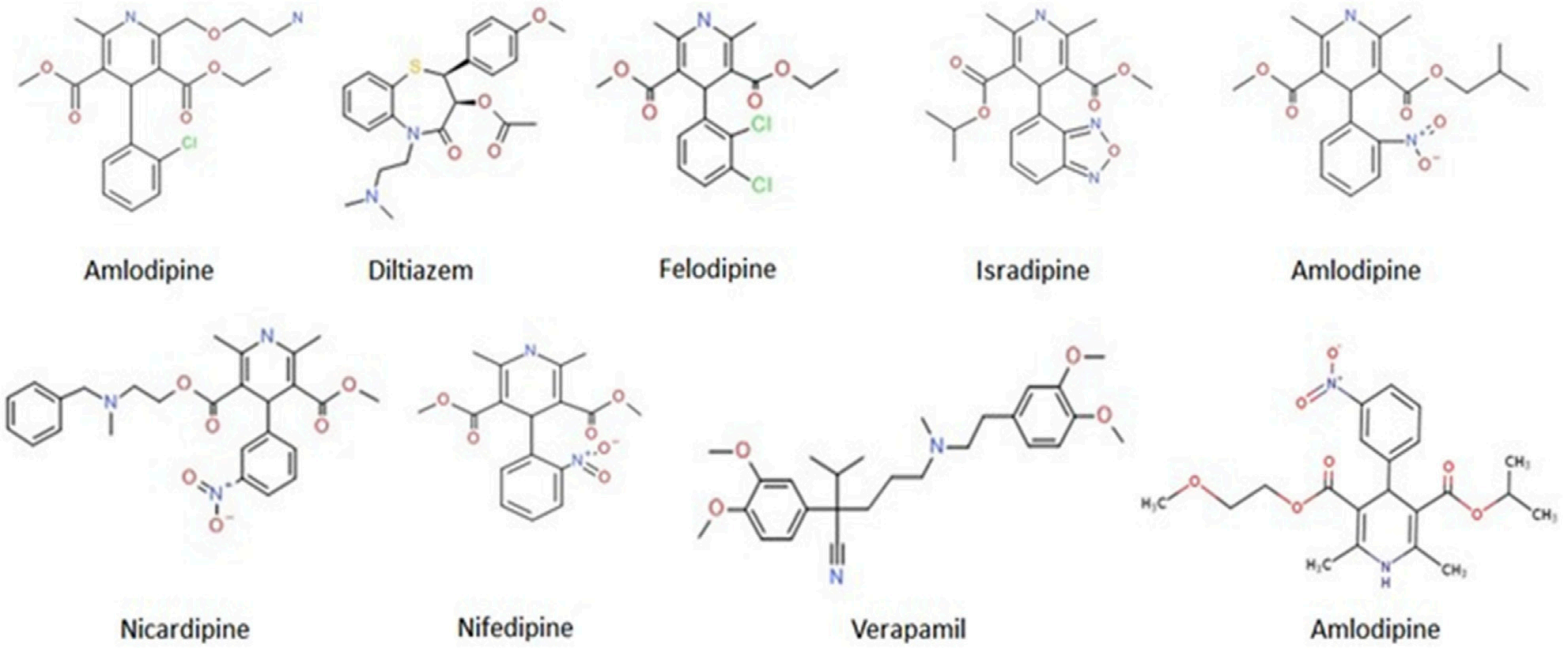
Molecular structures of the FDA-approved CCBs. Online source: https://pubchem.ncbi.nlm.nih.gov/compound.

Verapamil belongs to a class of CCBs, comprising drugs that mediate the relaxation of vascular and arterial smooth muscle cells to cause arterial vasodilation (Brozovich et al., 2016). Therefore, the CCBs are primarily prescribed for treating hypertension, arrhythmia, angina, and coronary artery disease (CAD) (Koracevic et al., 2022). Based on their chemical structure, CCBs are classified into two main classes: i) non-dihydropyridines, which include phenylalkylamines (verapamil) and benzothiazepines (diltiazem); and ii) dihydropyridines, which include amlodipine, bepridil, felodipine, isradipine, nicardipine, nifedipine, and nisoldipine (Figure 1). The mechanisms of action of both classes are different, each class raising concerns regarding its safety and efficacy (McDonagh et al., 2005). While, non-dihydropyridines target the heart muscles and regulate cardiac rhythm, the dihydropyridines target blood vessels and lower blood pressure (Opie, 1997).

At the cellular level, CCBs disrupt the influx of calcium ions across the cell membrane by blocking the L-type voltage-gate calcium ion channel (Shah et al., 2022). However, the site of interaction within the channel differs between these CCBs (Katz, 1986). Verapamil blocks the phenylalkylamine site and diltiazem blocks the benzothiazepine site, while other agents, such as amlodipine and nifedipine, bind to the 1,4 dihydropyridine site (National Institute of Diabetes and Digestive and Kidney Diseases and The, 2012). Unlike other CCBs, verapamil blocks both L-type and T-type calcium channels, thus possessing a higher affinity for depolarized channels than for resting channels (Johnson et al., 1996; Freeze et al., 2006; Bergson et al., 2011).

## 3 From hypertension to diabetes mellitus

In the mid-1970s, Dr. Willy J. Malaisse and colleagues established seminal studies delineating the role of verapamil in regulating pancreatic β-cell function using isolated pancreatic islets from rats. Their results showed that verapamil infusion caused a dose-related inhibition of glucose-induced insulin release in the isolated perfused rat pancreas (Devis et al., 1975). The degree of inhibition was challenged by the application of calcium agonists (Somers et al., 1976a; Somers et al., 1976b), suggesting that verapamil blocks the entry of calcium into the β-cell (Malaisse et al., 1977), and partially inhibits the insulin release (Somers et al., 1979). These observations were further supported using pharmacological approaches and genetically modified rodent models (Vasseur et al., 1987), and are reviewed by Tuluc et al. (2021).

Later, in 1992, Wei et al. provided the experimental evidence showing that verapamil protects the pancreatic β-cells from damage by exogenous toxins in rats (Wei et al., 1992). Their histological results revealed that pretreatment with verapamil enriched the number of secretory granules in β-cells of pancreatic islets in alloxan-injected rats (Wei et al., 1992). Similarly, verapamil pretreatment prevented alloxan-induced hyperglycemia in rats (Kim et al., 1994). In rats subjected to verapamil pretreatment, isolated pancreatic islets showed that in β-cells, the process of insulin secretion was unaltered, along with an absence of DNA strand fragmentation and reduced concentrations of calcium ions (Katsumata et al., 1988; Kim et al., 1994).

The above findings led researchers to further explore the effects of verapamil on T1D and T2D, using both *in vitro* and *in vivo* settings, along with clinical studies in humans. In the following sub-sections, we explore the current scenario of verapamil therapy in patients with T1D and T2D and overview the history of its clinical applications (summarized in Table 1).

**TABLE 1:** A summary of verapamil clinical studies measuring effects on blood glucose and the impact on pancreatic β-cells.

Year	References	Study design	Type of Diabetes	Treatment Dose and duration	Study Findings
T1D					
2018	Ovalle et al. (2018)	Phase 2 randomized, double-blind, placebo-controlled clinical trial	Recent-onset T1D	120–360 mg sustained-release once daily oral dose for 12 months	Adding oral verapamil once daily to a standard insulin regimen provides a safe and effective approach to promote and preserve the endogenous β-cell function, delay β-cell loss and disease progression, and reduce insulin requirements and hypoglycemic episodes in adults with recent-onset T1D
2022	Xu et al. (2022)	Phase 2 randomized, double-blind, placebo-controlled clinical trial	Recent-onset T1D	360 mg sustained-release daily oral dose for 12 months	Continuous oral verapamil administration in patients with T1D might delay the loss of β-cell function and reduce the exogenous insulin requirements, while the discontinuation of verapamil treatment might lead to disease progression
2023	Forlenza et al. (2023)	Multi-center randomized, double-blind clinical trial	Recent-onset T1D	Starting oral dose: 60,120 mg once daily. Maximum dose: 360 mg once daily for 52 weeks (The drug dose was dependent on each participant’s weight. The dose was escalated at 2- to 4-week intervals to a maximum dose for participants weighing more than 50 kg. The dose was held, decreased, or discontinued if adverse events occurred)	Elevated and sustained C-peptide levels in pediatric T1D patients treated with verapamil, demonstrating beneficial effects of verapamil on pancreatic β-cell function
2023	Xu et al. (2023)	Clinical trial	T1D	360 mg sustained release once daily for 12 months	A proteomics analysis of serum samples obtained from patients with T1D at the baseline and 1 year after verapamil or placebo treatment. Verapamil treatment has a beneficial effect on β-cells via the downregulation of both TXNIP and IGFBP3 and the increase in IGF-1 signaling
T2D					
1981	Andersson and Röjdmark (1981)	Placebo controlled clinical trial	T2D	10 mg iv, or 80 mg orally three times a day for 1 week	In T2D patients, verapamil improved oral glucose tolerance, regardless of the route of verapamil administration
1986	Röjdmark and Andersson (1986)	Placebo controlled clinical trial	T2D	80 mg orally three times a day for 1 week	Improved oral glucose tolerance in patients with type 2 diabetes without affecting insulin release by affecting the liver, leading to decreased hepatic glucose output, by regulating enzymes involved in gluconeogenetic and glycogenolytic processes
1989	Chellingsworth et al. (1989)	Placebo controlled clinical trial	T2D	120 mg orally two times a day for 1 month	Verapamil use had advantages over nifedipine and diltiazem, including an increase in fasting serum immunoreactive insulin in T2D hypertensives patients
1991	Busch Sørensen et al. (1991)	Single blind clinical trial	T2D	240 mg orally two times a day for 1 week	A brief verapamil administration decreased the fasting plasma glucose and glucose turn-over in T2D patients
2006	Cooper-Dehoff et al. (2006a)	Large-cohort multi-center studies (INVEST study)	T2D	120–480 mg/day orally (individualized dose based on the level of blood pressure), Average 2.7-year follow-up	Verapamil decreases the risk of T2D development in patients with CAD.
2009	Bakris et al. (2004), Cooper-DeHoff et al. (2009)	Large-cohort multi-center studies (INVEST study)	T2D	120–480 mg/day orally (individualized dose based on the level of blood pressure), Average 2.7-year follow-up	After 2–5 years of follow up, verapamil-treated participants enrolled in the INVEST trial were less likely to develop T2D
2009	Bakris et al. (2009)	Multicenter, prospective, randomized, open-label, blinded endpoint (PROBE) clinical Trial (STAR study)	New onset of T2D	180 mg/day orally for 52 weeks	Patients with metabolic syndrome were at a lower risk of developing T2D when treated with a combination of verapamil and trandolapril
2016	Khodneva et al. (2016)	Cohort study of community-dwelling middle-aged and older adults (REGARDS database)	T2D	As reported by the authors, the limitation of the study is the absence of the dose and duration of CCB treatment	A notable association between verapamil and lower fasting glucose levels in patients with diabetes enrolled in the REGARDS database
2017	Yin et al. (2017)	Retrospective population-based cohort study used Taiwan’s National Health Insurance Research Database from 2000 to 2011	T2D	This study was based on a national database, the use and duration of the drug was not mentioned	Verapamil use is associated with reduced incidence of newly diagnosed T2D
2021	Malayeri et al. (2021)	Randomized, double-blind, placebo-controlled clinical trial	T2D	120 mg/day orally for 90 days	A significant reduction in Hb1Ac levels in patients with T2D, following treatment with verapamil
2022	Wang et al. (2022)	Multicenter, Randomized controlled clinical trial in Taiwan and the United States (November 2017 to April 2020)	T2D	Three groups: 1) Three tables of 75 mg (225 mg) two times per day: total 450 mg/day, 2) Two tables of 75 mg (150 mg) two times per day: total 300 mg/day, and 3) one table of 75 mg two times per day: total 150 mg/day	Co-treatment with verapamil and metformin significantly improved glycemic control in patients with T2D by reducing glycated hemoglobin (HbA1c) and fasting plasma glucose levels

### 3.1 Verapamil protects β-cells and improves endogenous insulin in patients with T1D

In 2012, entailing to a decade-long quest for elucidating the role of TXNIP and related signaling pathways by Shalev group, Xu et al. reported positive effects of verapamil on β-cell survival and function, both in cell and rodent models, that were unprecedented (Xu et al., 2012). They showed that by reducing β-cell TXNIP expression, verapamil inhibited β-cell apoptosis, enhanced their survival and function, and rescued them from diabetes progression. This quest actually began in 2002, when this group performed a microarray analysis of the human pancreatic islets and identified TXNIP as a top-ranking glucose-induced gene (Shalev et al., 2002). They further found that the reduced TXNIP levels induced Akl/Bcl-xL signaling and inhibited β-cell loss, providing the evidence that TXNIP deficiency had β-cell protective effects (Chen et al., 2008). Next, they further found that CCBs, such as verapamil, reduced cardiac expression of TXNIP, thereby reducing apoptosis in cardiomyocytes (Chen et al., 2009). These cardiac effects of verapamil led onto determine if verapamil could influence β-cell TXNIP gene expression, and β-cell survival and protection. Taking into account verapamil’s effect on TXNIP expression in both cardiomyocytes and β-cells, it is now ascertained that this effect is not tissue-specific.

To translate these findings from bench to bedside, a Phase 2 randomized, double-blind, placebo-controlled clinical trial was conducted to investigate the safety and efficacy of oral verapamil as an add on to a standard insulin regimen in adult patients with recent-onset T1D (Ovalle et al., 2018). In this study, endogenous β-cell function was evaluated using a stimulated C-peptide area under the curve (AUC) determined during a 2-h mixed-meal tolerance test (MMTT) (Besser, 2013; Ovalle et al., 2018). This study concluded that adding oral verapamil once daily to a standard insulin regimen provided a safe and effective approach to promote and preserve the endogenous β-cell function, delayed β-cell loss and disease progression, and reduced insulin requirements and hypoglycemic episodes in adults with recent-onset T1D (Ovalle et al., 2018). Intrigued by their findings, this group further expanded their study and found that the continuous oral verapamil administration in patients with T1D might delay the loss of β-cell function and reduce the exogenous insulin requirements; while the discontinuation of verapamil treatment might lead to disease progression (Xu et al., 2022). It is noteworthy that in the study by Ovalle et al. (2018), although there was an increase in C peptide levels observed for the first 3 months in verapamil-treated patients, the level of C peptide deteriorated after 12 months of treatment, however, with a significantly slower disease progression, as compared to placebo-treated participants. This is not unexpected, given the continuous immunogenic damage to the β-cells in T1D; however, the C peptide levels were not adjusted to the autoantibody levels in this study, which could act as an indicator of disease progression. Therefore, future studies could focus on dose-adjustment of verapamil, depending on the level of autoantibodies or disease progression. Moreover, Xu et al. (2022) found that some patients responded better, supporting precision medicine as an avenue to explore in the future work involving verapamil as a diabetes treatment.

Recently, a multi-center randomized, double-blind clinical trial was conducted to determine the effect of verapamil on pancreatic β-cell function in children and adolescents, aged 7–17 years, with newly diagnosed T1D (Forlenza et al., 2023; McVean et al., 2023). These trials were unique and novel because they included young patients and the pathophysiology of T1D could be distinct in this population since the course of disease is often more aggressive in children than in adults. The results showed elevated and sustained C-peptide levels in pediatric T1D patients treated with verapamil, demonstrating the beneficial effects of verapamil on pancreatic β-cell function in pediatric patients with T1D (Forlenza et al., 2023; McVean et al., 2023). While no serious adverse events were reported in the study, some events such as psychiatric disorders were reported (Lefrandt et al., 2001). In these studies, although the researchers attempted to achieve the near-normoglycemic conditions, they observed clinically significant hyperglycemia. Additionally, the observed significant differences in C-peptide levels were not complemented with improvements in glycated hemoglobin (HbA1c), blood glucose levels, or insulin dose. This could be owing to their conservative approach as this was the first study investigating verapamil treatment in children (Forlenza et al., 2023), for whom the dual factors of weight- and symptom-based dose escalation were considered. Having said that, it would still be interesting to explore such young patient populations and decipher if verapamil would be a worthy treatment for them, which would get us a step closer to precision medicine.

### 3.2 Verapamil improves the glucometabolic response in patients with T2D

In the 1980s, Swedish investigators led several clinical trials exploring the effects of verapamil on glucose response and tolerance in healthy and T2D patients, respectively (Röjdmark et al., 1980; Andersson and Röjdmark, 1981; Röjdmark and Andersson, 1986). In healthy, fasting individuals, verapamil augmented glucose response upon intravenous glucagon administration with no changes in plasma insulin levels, suggesting that the drug had a direct effect on hepatic gluconeogenesis (Röjdmark et al., 1980). In T2D patients, verapamil improved oral glucose tolerance, regardless of the route of verapamil administration (Andersson and Röjdmark, 1981). Further clinical investigations revealed that verapamil affected the liver, leading to a decreased hepatic glucose output, by regulating enzymes involved in gluconeogenetic and glycogenolytic processes (Röjdmark and Andersson, 1986). Then followed an era of head-to-head clinical studies. A team of researchers studied the effect of CCBs, such as verapamil, diltiazem, nifedipine, and propranolol, on patients with hypertension and T2D. They revealed that verapamil had several advantages over nifedipine and diltiazem, including an increase in fasting serum immunoreactive insulin (Chellingsworth et al., 1989). By the onset of 1990s, another group reported that a brief verapamil administration decreased the fasting plasma glucose and glucose turn-over in patients with non-insulin dependent diabetes mellitus (NIDDM), also known as T2D (Busch Sørensen et al., 1991).

An overall improvement in glucose homeostasis after treatment with verapamil encouraged researchers and clinicians to design large-cohort multi-center studies in the following decades. In the early 2000s, a large, multi-center study was designed, called INternational VErapamil SR-Trandolapril STudy (INVEST), which included 22,576 predominantly elderly patients with an average 2.7-year of follow-up (Bakris et al., 2004; Cooper-DeHoff et al., 2009). The authors compared between a calcium antagonist-based therapeutic strategy, i.e., verapamil SR plus trandolapril, and a β blocker-based therapeutic strategy, i.e., atenolol plus hydrochlorothiazide, for the treatment of hypertension and prevention of cardiovascular outcomes in patients with coronary artery disease (CAD). They reported that verapamil decreased the risk of T2D development in patients with CAD (Cooper-Dehoff et al., 2006b), with no severe adverse events (Bakris et al., 2004). As expected, after 2–5 years of follow up, verapamil-treated participants enrolled in the INVEST trial were found to be less likely to develop diabetes (Cooper-DeHoff et al., 2009). In 2009, results of the Study of Trandolapril/Verapamil SR And Insulin Resistance (STAR) were published which investigated the effects of two fixed-dose combinations (trandolapril/verapamil SR and losartan/hydrochlorothiazide (L/H)) on glucose control and new-onset diabetes in patients with metabolic syndrome (MetS). This study suggested that patients with MetS might be at a lower risk of developing diabetes when treated with a combination of CCB (verapamil) and angiotensin-converting enzyme (ACE) inhibitor (trandolapril) (Bakris et al., 2009). In 2016, Khodneva et al. investigated the REasons for Geographic and Racial Differences in Stroke (REGARDS) database that included a national cohort of community-dwelling, middle-aged and older 4,978 adults with diabetes, enrolled between 2003 and 2007 in the United States (Khodneva et al., 2016). In this study, Khodneva et al. observed a notable association between verapamil and lower fasting glucose levels in this patient cohort (Khodneva et al., 2016). Similar observations were reported in patients with diabetes enrolled in Taiwan’s National Health Insurance Research Database (Yin et al., 2017) and in this retrospective population-based study, Yin et al. found that oral verapamil administration was associated with a decreased T2D incidence, with a more prominent effect observed in individuals aged >65 years (Yin et al., 2017).

In 2020, the first systematic review of its kind was published by Carnevale et al., wherein they investigated the impact of verapamil-based treatment on glucometabolic outcomes in patients with T2D. The authors studied 11 controlled clinical trials and concluded that verapamil treatment was associated with a significant decrease in plasma glucose levels. However, no such improvements in HbA1c levels were noted. Based on this review, the glucometabolic response induced by verapamil-based treatment remains unclear, possibly due to the high variability in sample size and study types, indicating the need for further experimental studies and clinical trials to clarify the role of verapamil in glucometabolic control (Carnovale et al., 2020). To investigate this annotation, an elegant study was conducted by Malayeri et al., who evaluated the efficacy and safety of oral verapamil in patients with T2D. This randomized, double-blind, placebo-controlled clinical trial showed a significant reduction in Hb1Ac levels in patients with T2D, following treatment with verapamil (Malayeri et al., 2021). It is noteworthy that co-treatment with verapamil and metformin significantly improved the glycemic control in patients with T2D by reducing HbA1c and fasting plasma glucose levels (Wang et al., 2022). Applying to precision medicine, an innovative approach to tailoring disease prevention and treatment would be by taking into account the differences in genes, ethnicity, environment, and lifestyle, in order to provide a more elaborative data regarding efficacy and safety of verapamil in maintaining HbA1c level and general blood glucose management, especially in populations with diverse attributes.

## 4 Molecular aspects of Verapamil action

Researchers around the world embarked on their quest to decipher how verapamil was able to render protection to β-cells, enhance and preserve β-cell function, and reduce dependency on exogenous insulin. A study published in the early 2000s showed that TXNIP, a key redox regulator, was the most highly expressed in β-cells under diabetic conditions (Shalev et al., 2002). TXNIP inhibits the action of thioredoxin via regulation of the redox metabolism and modulates the cellular redox state and promotes oxidative stress (Coucha et al., 2019). Across that decade, key studies on TXNIP revealed that its overexpression induces apoptosis in β-cells (Minn et al., 2005) and that TXNIP acts as a critical mediator of glucotoxicity-induced β-cell apoptosis (Chen et al., 2008). The latter study provided strong evidence that TXNIP might play a key role in diabetes progression and the associated loss of β-cell mass. In 2010, Zhou and colleagues found that TXNIP induced inflammasome activation, resulting in the production of interleukin (IL)-1β, a key cytokine involved in T1D, and that TXNIP downregulation could have anti-inflammatory effects (Zhou et al., 2010). Xu. et al., in 2012, further validated decades-long research and showed that verapamil lowered the TXNIP expression in rat INS-1 cells, as well as in human and C57BL/6 mice islets (Xu et al., 2012). Moreover, they showed that through inhibiting TXNIP expression, verapamil treatment significantly reduced β-cell apoptosis, promoted β-cell survival and glucose homeostasis, prevented diabetes progression or delayed diabetes development, and could even avert diabetes in streptozotocin-treated mice (Xu et al., 2012). Reportedly, these results were owing to the regulation of calcineurin signaling and increase in the nuclear exclusion of the carbohydrate response element–binding protein (ChREBP) which in turn downregulates TXNIP-mediated ChREBP activation, leading to reduction in the TXNIP expression and β-cell apoptosis (Xu et al., 2012). These results are supported by a recent study conducted by Malayeri et al., in 2021, which showed that TXNIP transcripts expression was relatively lower in blood samples from patients treated with verapamil, as compared to placebo-treated cohort (Malayeri et al., 2021).

In 2022, researchers ventured to decipher the exact changes verapamil elicits in patients with T1D and the longevity of these changes (Xu et al., 2022). Results of their proteomics analysis revealed that verapamil treatment significantly altered 53 proteins, including the downregulation of proinflammatory IL-21 and T-follicular helper (Tfh) cell markers, indicating that verapamil regulates the innate and adaptive immune responses. Notably, the neuroendocrine chromogranin A (CHGA), a T1D-autoantigen, emerged as the top serum protein that was normalized post-treatment with verapamil. CHGA is localized in β-cell secretory granules (Bearrows et al., 2019); therefore, a normalization of its circulating levels may reflect restoration in β-cell integrity, and it may serve as a longitudinal biomarker for monitoring therapeutic success (Xu et al., 2022). The transcriptomics data revealed downregulation of the MHC class I and II genes, proinflammatory cytokines, and oxidative stress mediators as well as upregulation of the anti-apoptotic genes, indicating that verapamil treatment may play a potential role in the regulation of T1D autoimmunity (Xu et al., 2022). Of particular interest, the expression of *TXNIP* was consistently decreased in response to verapamil. In contrast, a significant upregulation of thioredoxin reductase (*TXNRD1*) and sulfiredoxin (*SRXN1*) expression was observed and since these genes preserve the cellular redox potential, their upregulation may explain the protective effects of verapamil (Xu et al., 2022).

Most recently, Xu et al. conducted a global proteomics analysis of serum samples obtained from patients with T1D at the baseline and 1 year after verapamil or placebo treatment revealed that verapamil blunted the decrease of insulin-like growth factor (IGF-1) protein expression by increasing β-cell IGF-1 signaling and activating the IGF-1 receptor. IGF-binding protein 3 (IGFBP3) blocks the downstream signaling of IGF-1 by binding to it and results in β-cell apoptosis and impaired glucose homeostasis (Xu et al., 2023). The researchers also found that TXNIP promotes IGFBP3 expression and inhibits the activation of IGF-1 receptor. Taken together, verapamil has a beneficial effect on β-cells via the downregulation of both TXNIP and IGFBP3 and the increase in IGF-1 signaling (Xu et al., 2023).

**FIGURE 2:**
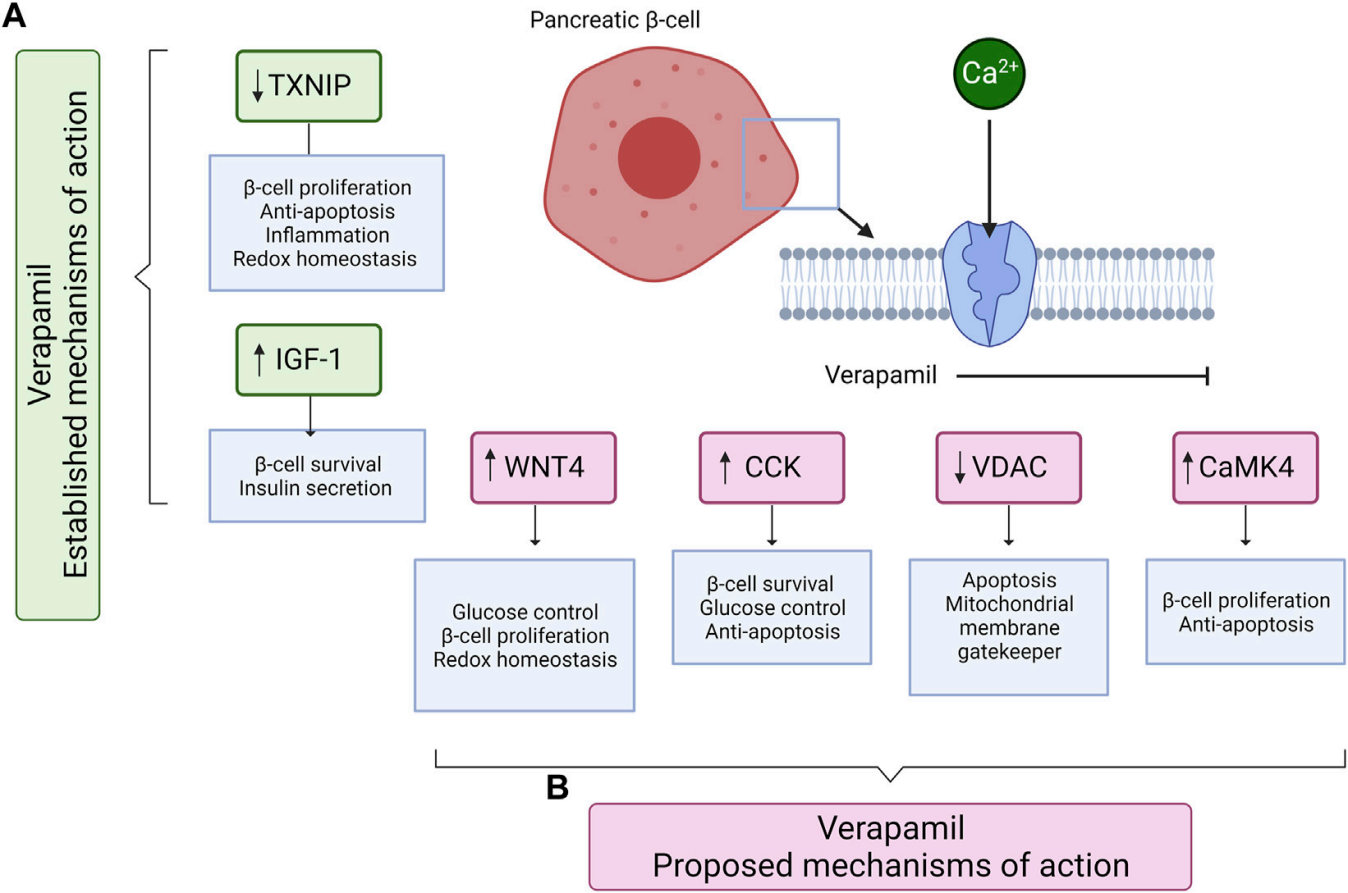
A schematic diagram shows the known and proposed mechanisms of action of verapamil, mediating the survival, proliferation, and protection of pancreatic β-cells. **(A)** The established mechanisms of action of verapamil include the reduction in TXNIP and increase in IGF-1 expression. **(B)** The proposed mechanisms of action of verapamil involve the increased expression of WNT4, CCK, and CaMK4 and the reduced expression of VDAC proteins. Up (↑) and down (↓) arrows represent the increased and decreased expression, respectively. TXNIP: Thioredoxin-interacting protein; IGF-1: Insulin-like growth factor 1; WNT4: Wnt family member 4; CCK: Cholecystokinin; VDAC: Voltage-dependent anion channels; CaMK4: Calcium/calmodulin-dependent protein kinase type IV. The illustration was created using Biorender.com.

Alterations in the expression of the above-mentioned factors led our team to investigate the role of verapamil in *in vitro* and *in vivo* models (manuscript under review). Regarding analyses of the proteomic and transcriptomic landscapes of verapamil-treated mouse insulinoma MIN-6 β-cell line model, our group found the altered mRNA and/or protein expressions of the factors involved in pathways pertaining to β-cell survival, function, and proliferation (Arefanian et al., 2023a; Arefanian et al., 2023b; Arefanian et al., 2023c). Based on the results of our analyses, combined with the previously established evidence, we present the plausible mechanisms of action of verapamil via which it might offer protection to β-cells (through anti-apoptotic pathways) as well as enhance β-cell mass and improve glycemic control (through pathways promoting cellular proliferation, Figure 2).

The most dominant upregulated factor in our analysis, at both the mRNA and protein levels was the glucagon-like peptide-1 (GLP-1)-induced incretin, cholecystokinin (CCK). CCK is known to play a role in preserving β-cell mass with increasing age and providing protection to β-cells against apoptosis induced by streptozotocin and cytokines (Lavine et al., 2015). Our preliminary data is the first to show that in β-cells, verapamil induces the expression of CCK and may perhaps protect the cell against apoptosis and ameliorate blood glucose control (Arefanian et al., 2023a; Arefanian et al., 2023b; Arefanian et al., 2023c). The voltage-dependent anion channels (VDACs) are another group of proteins known for their abundance in the outer mitochondrial membrane and for their major role in apoptotic signaling (Abu-Hamad et al., 2006; De Marchi et al., 2021). Previous studies have established the role of VDAC1 overexpression as an inducer of apoptotic death in response to high glucose conditions (Ahmed et al., 2010). Our analyses found that VDAC 1 and 2 are downregulated when exposed to verapamil treatment, possibly, at least partly, explaining the protective effect of verapamil (Arefanian et al., 2023c). Moreover, considering that VDAC1 is highly expressed in states of glucotoxicity, such as in pre-diabetes patients, verapamil could prevent the development and progression of diabetes, as also shown in previous clinical studies. CaMK4 is another key player in the apoptotic pathway that mediates *Irs2* expression by activating the cAMP response element binding protein (CREB) (Liu et al., 2020). It has been shown that *Irs2* knockout increases β-cell apoptosis and reduces β-cell mass; further, upregulated gene and protein expression of CaMK4, via the glucose/CaMK4/*Irs2* cascade, regulates the survival and proliferation of β-cells by inhibiting apoptosis and stimulating cell proliferation (Arefanian et al., 2023a; Arefanian et al., 2023c). Lastly, we found upregulated levels of Wnt4, a heterogeneously expressed ligand in β-cells known to activate calcium signaling, lowering blood glucose levels, and regulating redox homeostasis (Katsumoto et al., 2022). Wnt4 is known to play a pivotal role in β-cell proliferation and ameliorating the insulin secretory response (Kurita et al., 2019), along with an upregulation of genes associated with β-cell function and maturation (Katsumoto et al., 2022). We also found higher basal and maximal mitochondrial respiration levels in verapamil-treated cells, which we propose to be attributed to Wnt4 overexpression as it has been shown to upregulate the expression of genes inducing higher mitochondrial abundance (Katsumoto et al., 2022).

## 5 Conclusion and future perspectives

This review highlights how verapamil, a CCB used to treat cardiac pathologies, attracted attention as a drug having potential benefits for individuals with diabetes. The proposed mechanisms provide insights into the fundamental pathways that can be targeted to augment verapamil benefits or further develop more effective novel therapies. There is a growing need for T1D interventional trials, aiming at the functional β-cell mass restoration for better management and disease prevention. Daily oral verapamil administration along with conventional insulin regimen emerges as a promising approach, with no severe adverse events reported. Inclusion of immunomodulatory interventions in such trials is highly desirable. A close electronic health data monitoring in multi-center, multi-ethnic, large cohorts may help elucidate whether verapamil effectively prevents diabetes progression.

Moreover, the current clinical trials have mainly focused on new-onset diabetes, while it would be interesting to further see whether verapamil also benefits individuals with a long history of diabetes or those with pre-diabetes or with MetS (known as high-risk populations). Given that using verapamil in clinical trials dawns as a new era in T1D therapy, researchers working in the field are encouraged to explore its mechanism of action through long-term follow-up studies. In view of the limitations of past trials, pharmacogenetics studies prior to start of a clinical trial may ensure better outcomes, upholding verapamil as a potential candidate for precision medicine. We also believe that the involvement of international societies and organizations would be highly beneficial in implementing guidelines and consensuses for the safer practice of verapamil therapy in diabetes.
